# Field evaluation of synthetic and neem-derived alternative insecticides in developing action thresholds against cauliflower pests

**DOI:** 10.1038/s41598-019-44080-y

**Published:** 2019-05-22

**Authors:** Farhan Mahmood Shah, Muhammad Razaq, Qasim Ali, Sarfraz Ali Shad, Muhammad Aslam, Ian C. W. Hardy

**Affiliations:** 1 Department of Entomology, Faculty of Agricultural Sciences and Technology, Bahauddin Zakariya University, Multan, 60000 Pakistan; 2 Faculty of Forestry and Wood Sciences, Czech University of Life Sciences, Kamýcká, 1176 Czech Republic; 3 COMSATS Institute of Information Technology, Vehari, Pakistan; 4 School of Biosciences, University of Nottingham, Sutton Bonington Campus, Loughborough, UK

**Keywords:** Agroecology, Entomology

## Abstract

Synthetic chemical pesticides can enhance crop yields but also have undesired effects. Alternative ‘botanical insecticides’ may also have non-target effects on pollinators and biocontrol services. Employing action thresholds (ATs) can reduce pesticide (whether synthetic or botanical) use compared to fixed-interval applications. Here the azadirachtin-based botanical formulation NeemAzal and a neem seed extract (NSE) were evaluated in field spraying trials alongside commonly-used synthetics (Voliam Flexi [chlorentraniliprole plus thiamethoxam] and imidacloprid) in developing ATs for the regular and cosmopolitan cauliflower pests *Brevicoryne brassicae*, *Plutella xylostella* and *Spodoptera litura*. We considered the size of the *S. litura* larvae infesting the crop in order to derive ATs. ATs per plant were higher for NeemAzal (0.55 larvae for *P. xylostella* and 3 larvae for large-sized *S. litura*) than for Voliam Flexi (0.30 larvae for *P. xylostella* and 0.80 larvae for *S. litura*) but were similar for *B. brassicae* (50 individuals). Higher ATs when using azadirachtin were associated with the diverse modes of action of botanicals, for instance NeemAzal and NSE deterred oviposition of *S. litura*. Although the exact values of ATs are likely to have regional limits, our approach can be applied for determining ATs against common lepidopteran and aphid pests in many other vegetable crop agro-ecosystems.

## Introduction

Organic insecticides were introduced into agricultural production systems around eighty years ago. Although they delivered substantial suppression of pests both in agriculture and in public health, over-reliance on these chemicals subsequently generated health and environmental challenges^1^, and became the motivation for the development of integrated pest management (IPM) shortly after the second world war^2^. IPM relies on decision-making tools to promote the judicious use of pesticides. One such decision-making tool is the economic injury level (EIL), the lowest pest density capable of causing economic damage. When the pest has attained EIL, the cost of control and damage incurred are equal^3^. The relationship between the cost of control and damage is well established using a robust model (EIL = C/VDIK, where C = management cost per production unit, V = market value per production unit, D = damage per unit injury, I = injury per pest equivalent and K = proportional reduction in injury with management)^4^. The EIL is necessary for understanding the relationship between the pest and host crop, and thus is fundamental to establishment of the economic threshold (ET; the pest density that justifies treatment aimed at preventing an increasing pest population from reaching EIL). The inverse relationship between the EIL and crop value, V, indicates that EILs will be influenced by price^5^; as some cropping systems have unpredictable future prices^6^, and, as several further biotic and abiotic factors influence ETs, the establishment of ETs can be complex^6^.

The action threshold (AT) is another widely accepted decision-making tool in pest management^7^. It may be defined as the number of pests, or level of pest damage, at which control should be applied to prevent damage from exceeding tolerable levels. Although the derivation of ATs is not typically through EIL models^6^ and does not explicitly incorporate estimates of crop value or control costs^8^, ATs and ETs are often referred to synonymously and their recommendations are used alike in pest management decisions^6^. Like ETs, ATs also represent a quantifiable relationship between the pest species present and their damage to the economic value of the crop but quantifying such relationships is less complex^6^. Action thresholds can be developed by trialing a range of candidate ATs and subsequently adopting those that perform best and also by using prior experience of the crop-pest relationship^6^. Due to their relative ease of derivation, ATs are adaptable and can be adjusted for planting dates^7^, varieties, environmental conditions^6^ and biocontrol services^9^. ATs have been used successfully for the management of many agricultural and horticultural pests^8,10^ resulting in reduced use of agrochemicals^11^. Their use is likely to be well suited to vegetable production systems as these often have unpredictable future crop prices, particularly in developing countries.

Cruciferous vegetable crops, including cauliflower, *Brassica oleracea* var. *botrytis*, are grown in almost all of the world’s agricultural areas. Some of the most serious cauliflower pests in many countries, including Pakistan^12–14^, are the insects *Plutella xylostella* (L.), *Spodoptera litura* (F.) (Lepidoptera: Noctuidae) and *Brevicoryne brassicae* (L.) (Hemiptera: Aphididae). Aphids damage the plants directly by sucking phloem sap and indirectly by releasing honeydew, which subsequently provides a medium for fungal growth, interfering with photosynthetic and respirational activities of plant, and by influencing the spread and transmission of pathogens, such as cauliflower mosaic virus^15^. Lepidopterans chew holes in the leaves, reducing photosynthetic capacity and thereby affecting the quantity marketable produce (i.e. weight and diameter) while frass-induced cosmetic changes qualitatively decrease market value. These problems generate immense pressure on growers to protect yield losses.

Growers usually prefer synthetic pesticides for controlling pests due to their rapid effects. Reliance on synthetics is most  extensive in less developed countries due to their easy availability. Insecticides are usually applied on a regular basis, such as 2 applications per week against *P. xylostella*^16^. Farmers typically continue to apply insecticides at the fruiting stage, even though this could increase the absorption of toxins^17^. Multiple applications of insecticides, alone or as mixtures, can also negatively affect non-target arthropods, such as beneficial natural enemies (predators and parasitoids)^18,19^, and could select for multiple forms of pest resistance^20,21^. The deployment of synthetic pesticides should proceed using ATs to warrant their judicious use and to minimize their undesired effects. In the cauliflower agro-ecosystem, ATs have been developed for *P. xylostella* using synthetic pesticides^17,18^ but none have been developed for the control of *S. litura* or *B. brassicae*. There has also been almost no prior development of ATs using non-synthetic alternatives such as biopesticides based on plant-based products (botanicals)^11^.

Botanicals constitute around 5.6% of all biopesticides (and <0.05% of all pesticides) applied worldwide, although their usage appears to be increasing in China, Latin America and Africa^22^, regions in which socio-economic conditions have led to some of the worst examples of human poisoning and environmental contamination^23,24^. Botanicals could be especially valuable in developing countries^25,26^ where the source plant species are often locally abundant and accessible and the preparation of extracts is inexpensive^22,27^. For instance, seeds and other parts of the neem tree (*Azadirachta indica*, A. Juss. L., family Meliaceae) native to the Indian subcontinent, contain a major active ingredient, azadirachtin that is known to adversely affect oviposition, feeding and growth of over 540 pest insect species^28,29^. Neem formulations (containing pure active ingredient) and seed aqueous extracts (blends of active substances), have the potential to be used in the management of various agricultural and horticultural pests^30,31^.

Interest in the botanical pesticides as alternative to synthetics was developed mainly due to properties such as low human toxicity, easy degradation and environmental safety^32,33^. Moreover, they can exhibit various modes of action against target pests, which favours their adoption in IPM as a resistance management strategy. However, biopesticides may induce sub-lethal behavioural and physiological effects in non-target beneficial organisms, such as pollinators and biocontrol agents of ecosystems: in addition to direct exposure, pollinators are exposed to botanical residues by pollen, nectar and honey that often contain residues of botanicals, another cause for declining bee populations^34^. In one study, azadirachtin and imidacloprid were found to be equally toxic to bees^35^. The development of ATs is one route towards the cautious inclusion of botanicals into pest management programs.

Here we evalute the species compostition of insect pests of field-grown cauliflowers in Pakistan where cauliflower is grown by both commercial and subsistance farmers. There is very little tolerance to insect infestation when califlowers are sold in local markets, thus synthetic pesticides are the most widely adopted pest-control measure. Given their deleterious effects, developing strategies to minimize and/or replace the use of synthetic chemicals in crops, especially vegetables that are consumed fresh, is an important pest management goal. We report on season-long field experiments, carried out in two major cauliflower-growing districts. Considerations include variation in planting dates, monitoring of pest numbers and phenology and criteria for crop marketablity and value. We employ these considerations to develop action thresholds for the major pests *P. xylostella*, *S. litura* and *B. brassicae*, using both synthetic and neem-derived insecticides.

## Results

The guild of insect pests associated with cauliflowers included one species of aphid, *B. brassicae*, and five species of lepidopterans. Overall, *Spodoptera litura* was the most abundant lepidopteran followed by *P. xylostella* but, when present, the numbers of aphids exceeded the numbers of lepidopterans by at least one order of magnitude (Supplementary Table S1). Thus, these species were our major focus for developing action thresholds.

**Figure 1:**
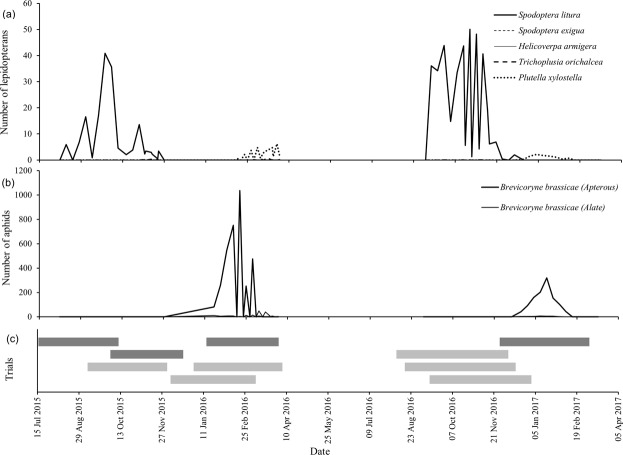
Overview of seasonal dynamics of insect pests and the timing of experimental trials. Mean numbers of pests present across untreated control plots, for all trials running at each given date, are shown from the start of the first trial until the end of the final trial. (**a**) Lepidopterans. (**b**) Aphids. (**c**) Timing of trials: Dark bars, Multan trials; Light bars, Bahawalpur trials (see also Table 1).

In control plots, with no insecticide applied, *S. litura* was recorded between early-August until the end of November, with peak abundancde around the end of September each year (Fig. 1a), while *S. exigua*, *H. armigera* and *T. orichalcea* were recorded for a shorter periods in November 2015 and October 2016 (Fig. 1a). *Brevicoryne brassicae, P. xylostella* and *T. orichalcea* were recorded between the end of December and the end of March each year (Fig. 1a,b). The majority of *B. brassicae* observed were apterous (Fig. 1b).

### Effect of sowing date on overall pest abundance

The overall composition of pests (species and numbers) present across all treatments (see Table 1 for treatment details within trials) was affected by sowing date at both sites (MANOVAs: Table 2, Fig. 1c). In terms of individual pest species, seasonal totals of *B. brassicae* were not affected by sowing date but seasonal totals of *S. litura* and *P. xylostella* differed significantly according to the time of cauliflower planting (Table 2). Seasonal totals of *T. orichalcea*, *H. armigera* and *S. exigua* individuals that were present only in low numbers (Supplementary Table S1), did not differ consitently according to sowing date, with significant effects only at Bahawalpur (Table 2).

**Table 1:** Summary of trials on cauliflower.

Planting date^a^	Location^b^	Cultivar^c^	Sowing within rows^d^	Insecticidal treatments within each trial^e^							
				Control	VF-5	VF-10	VF-15	NA-7	NA-14	NSE-7	NSE-14
16^th^ July 2015	Multan (MK)	Sathra	Single	⦁	⦁	⦁	⦁	⦁	—	⦁	—
2^nd^ October 2015	Multan (MK)	White Excel	Double	⦁	⦁	⦁	⦁	⦁	—	⦁	—
19^th^ January 2016	Multan (MK)	Twingo	Double	⦁	⦁	⦁	⦁	⦁	⦁	⦁	⦁
2^nd^ December 2016^f^	Multan (BZU)	Smilla	Double	⦁	⦁	⦁	⦁	⦁	—	⦁	—
12^th^ September 2015	Bahawalpur	5340	Double	⦁	⦁	⦁	⦁	⦁	—	⦁	—
11^th^ December 2015^g^	Bahawalpur	Smilla	Double	⦁	—	—	—	⦁	⦁	⦁	⦁
5^th^ January 2016^f^	Bahawalpur	Hansa	Double	⦁	⦁	⦁	⦁	⦁	⦁	⦁	⦁
12^th^ August 2016	Bahawalpur	Sathra	Single	⦁	⦁	⦁	⦁	⦁	—	⦁	—
21^st^ August 2016	Bahawalpur	Sathra	Single	⦁	⦁	⦁	⦁	⦁	—	⦁	—
17^th^ September 2016	Bahawalpur	White Excel	Double	⦁	⦁	⦁	⦁	⦁	—	⦁	—

^a^Two further crops were planted in Multan on 3^rd^ July 2016 and 3^rd^ January 2017 but could not continue due to extremely low pest numbers in the first and flooding of seedlings in the second.

^b^MK = Moza Kayaanpur, BZU = Bahauddin Zakariya University.

^c^Cultivars planted at a given time of year were the varieties favoured by local farmers: variation in cultivar and in sowing date are thus largely confounded.

^d^Sathra was planted singly due to being a large-plant cultivar.

^e^Control = replicates not sprayed with any insecticides; VF = Voliam Flexi, replicates sprayed every 5^th^, 10^th^ or 15^th^ day; NA = NeemAzal, sprayed every 7^th^ or 14^th^ day, NSE = Neem seed extract, sprayed every 7^th^ or 14^th^ day. (⦁) = treatment included in trial, (—) = treatment not included in trial.

^f^In these trials aphids and lepidopterans appeared simultaneously, so plots which were scheduled to be sprayed with VF (against lepidopterans) every 5^th^, 10^th^ or 15^th^ day were also sprayed with imidacloprid (against aphids) every 7^th^, 14^th^ or 21^st^ day, respectively.

^g^In this trial aphids but no lepidopterans were present, so plots which had been scheduled to be sprayed with VF every 5^th^, 10^th^ or 15^th^ day were instead sprayed with imidacloprid every 7^th^, 14^th^ or 21^st^ day, respectively.

**Table 2:** Effects of insecticide treatments and sowing date^†^ on the total numbers of pests observed.

	Multan												Bahawalpur											
	2015–16						2016–17						2015–16						2016–17					
	Insecticide			Sowing date			Insecticide			Sowing date			Insecticide			Sowing date			Insecticide			Sowing date		
**ANCOVAs**	*F*	*df*	*P*	*F*	*df*	*P*	*F*	*df*	*P*	*F*	*df*	*P*	*F*	*df*	*P*	*F*	*df*	*P*	*F*	*df*	*P*	*F*	*df*	*P*
**Species**																								
*Spodoptera litura*	2.72	7,49	0.018	29.32	1,49	<0.001*	—	—	—	—	—	—	1.99	7,55	0.073	36.43	1,55	<0.001*	13.46	5,45	<0.001*	888.85	1,45	<0.001*
*Spodoptera exigua*	0.30	7,49	0.948	0.31	1,49	0.581	—	—	—	—	—	—	—	—	—	—	—	—	0.42	5,45	0.834	59.95	1,45	<0.001*
*Helicoverpa armigera*	0.68	7,49	0.685	0.33	1,49	0.571	—	—	—	—	—	—	—	—	—	—	—	—	6.74	5,45	<0.001*	5.36	1,45	0.02
*Plutella xylostella*	5.34	7,49	<0.001*	47.46	1,49	<0.001*	89.44	5,10	<0.001*	—	—	—	1.37	7,55	0.238	23.29	1,55	<0.001*	—	—	—	—	—	—
*Trichoplusia orichalcea*	1.00	7,49	0.443	0.18	1,49	0.670	—	—	—	—	—	—	1.43	7,55	0.211	20.90	1,55	<0.001*	0.98	5,45	0.439	2.92	1,45	0.09
*Brevicoryne brassicae*	—	—	—	—	—	—	228.90	5,10	<0.001*	—	—	—	2.42	7,55	0.031	2.07	1,55	0.156	—	—	—	—	—	—
**MANOVA**																								
Wilks’ λ,	0.094			0.420			<0.001			—			0.157			0.381			0.240			0.044		
Rao *F*_*(df)*_	4.11_(35,192)_			12.42_(5,45)_			46.07_(10,18)_			—			4.53_(28,189)_			21.07_(4,52)_			3.77_(20,140)_			224.54_(4,42)_		
*P*	<0.001			<0.001			<0.001			—			<0.001			<0.001			<0.001			<0.001		

^†^Effects of sowing date could not be evaluated in 2016–17 in Multan due to two trials being abandoned (Table 1); when evaluated, comparisons were across the first three plantings at each site in each year. Date effects could also be due to within-season variation in cultivars (Table 1).

*Because several ANCOVA tests were carried within years and sites we adjusted the significance criterion according to the Bonferroni procedure by dividing the significance criterion (0.05) by the number of species present at each site in each year. *P*-values less than these adjusted values are indicated with an asterisk.

### Effect of insecticide treatment on overall pest abundance

Insecticide treatment significantly affected the composition of pests present in at each site and in each year (MANOVAs: Table 2); in all cases, pests were more abundant in control plots than in plots treated with insecticide (Supplementary Fig. S1). Plots sprayed every 5^th^ or 10^th^ day with Voliam Flexi, and plots sprayed weekly with NeemAzal, frequently had lower pest numbers than the other insecticide treatments (Supplementary Fig. S1).

For individual pest species, insecticide treatment affected significantly the total numbers of *S. litura* and *P. xylostella* in 2015–6 and *P. xylostella* and *B. brassicae* during 2016–17 at Multan. At Bahawalpur, *S. litura* and *H. armigera* numbers were affected by insecticide treatment in 2016–17 (Table 2).

### Effect of insecticides on weekly pest abundance

**Figure 2:**
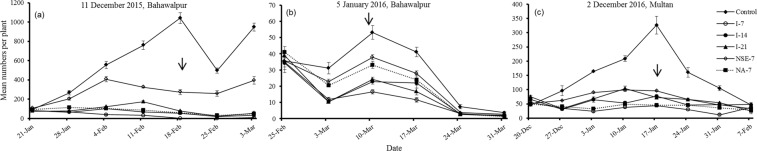
Effect of insecticide treatments on the weekly (mean ± SE) numbers of *B. brassicae* following different planting dates. Control = no insecticidal application, I-7 = imidacloprid application every 7^th^ day, I-14 = imidacloprid application every 14^th^ day, I-21 = imidacloprid application every 21^st^ day; NA-7 = NeemAzal application every 7^th^ day, NSE-7 = Neem seed extract application every 7^th^ day. Arrows indicate when the pest population reached a peak in the untreated plots. (Note the differing y-axis scales).

**Figure 3:**
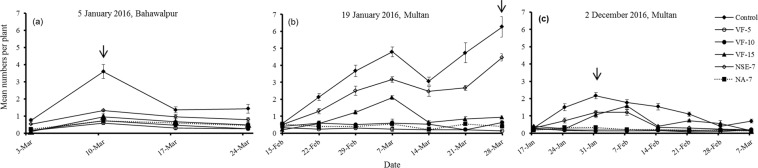
Effect of insecticide treatments on the weekly (mean ± SE) numbers of *P. xylostella* following different planting dates. Control = no insecticidal application, VF-5 = Voliam Flexi application every 5^th^ day, VF-10 = Voliam Flexi application every 10th day, VF-15 = Voliam Flexi application every 15^th^ day, NA-7 = NeemAzal application every 7^th^ day, NSE-7 = Neem seed extract application every 7^th^ day. Arrows indicate when the pest population reached a peak in the untreated plots.

**Figure 4:**
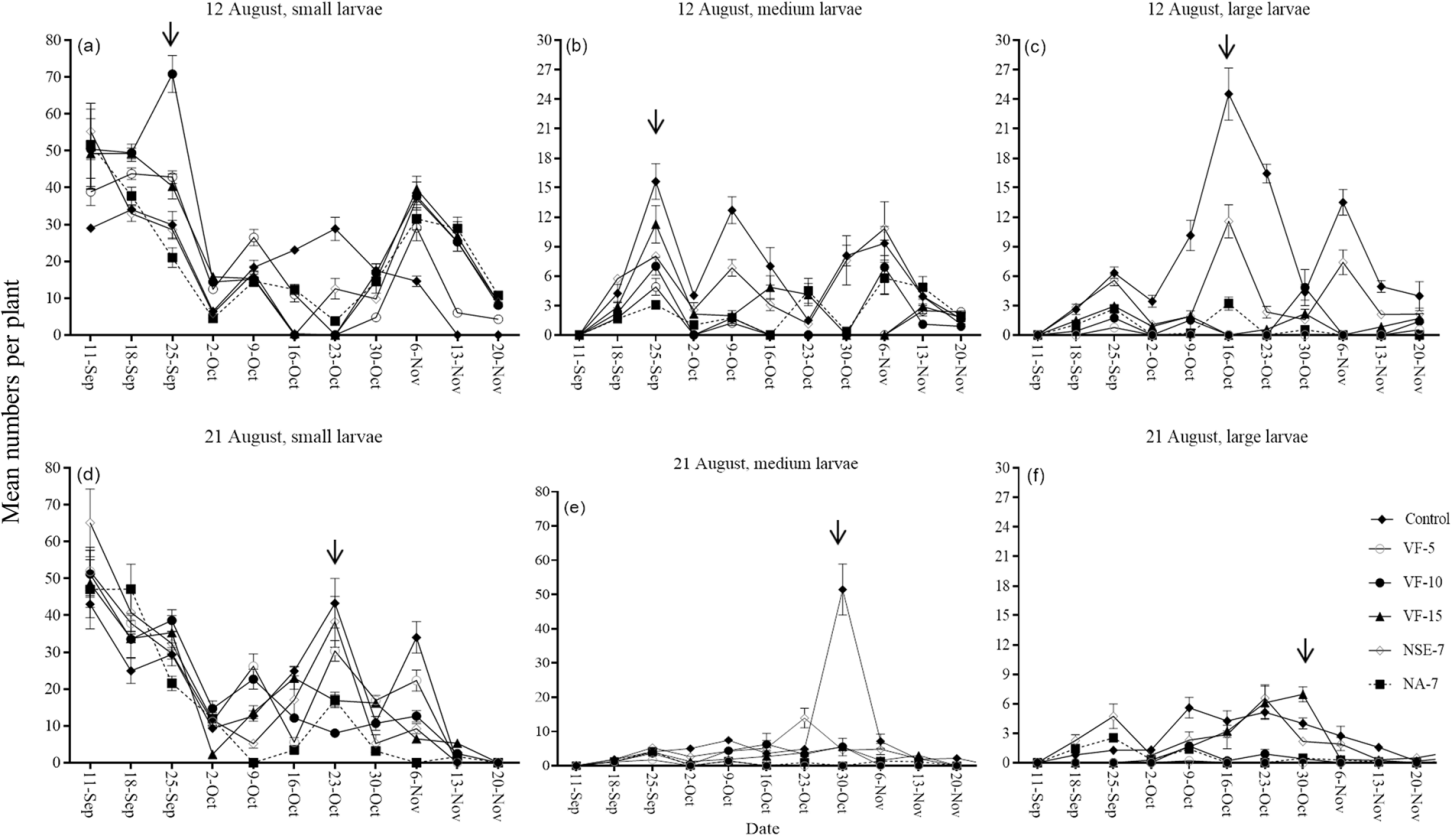
Effect of insecticide treatments on weekly (mean ± SE) numbers of small (<1 cm), medium (1–2 cm) and large (>2 cm) sized *S. litura* larvae following different planting dates. Control = no insecticidal application, VF-5 = Voliam Flexi application every 5^th^ day, VF-10 = Voliam Flexi application every 10th day, VF-15 = Voliam Flexi application every 15^th^ day, NA-7 = NeemAzal application every 7^th^ day, NSE-7 = Neem seed extract application every 7^th^ day. Arrows indicate when the pest population reached a peak in the untreated plots. (Note the differing y-axis scales).

Insecticide treatment, sampling time and their interaction typically affected the numbers of *B. brassicae*, *P. xylostella*, and the overall numbers of *S. litura* present (repeated measures ANOVAs, Table 3, Figs 2–4).

**Table 3:** Effects of insecticide treatments on weekly numbers of pests observed (Repeated measures ANOVA).

Species	Date	Site	Insecticide			Sample time			Insecticide × sample time interaction		
			*F*	*df*	*P*	*F*	*df*	*P*	*F*	*df*	*P*
*Spodoptera litura* (all larval sizes)	16^th^ July 2015	Multan	294.67	5,10	<0.001	341.23	9,108	<0.001	30.62	45,108	<0.001
	12^th^ September 2015^‡^	Bahawalpur	9.81	5	0.081^NS^	—	—	—	—	—	—
	2^nd^ October 2015^‡^	Multan	10.69	5	0.058^NS^	—	—	—	—	—	—
	12^th^ August 2016	Bahawalpur	58.52	5,10	<0.001	257.34	10,120	<0.001	37.96	50,120	<0.001
	21^st^ August 2016	Bahawalpur	158.07	5,10	<0.001	707.59	11,132	<0.001	23.51	55,132	<0.001
	17^th^ September 2016^‡^	Bahawalpur	15.00	5	0.010^†^	—	—	—	—	—	—
*Spodoptera exigua*	2^nd^ October 2015	Multan	0.87	5,10	0.535^NS^	53.01	2,24	<0.001	0.68	10,24	0.645^NS^
	17^th^ September 2016	Bahawalpur	0.36	5,10	0.868^NS^	0.82	3,36	0.466^NS^	1.23	15,36	0.315^NS^
*Helicoverpa armigera*	2^nd^ October 2015^‡^	Multan	12.33	5	0.030^†^	—	—	—	—	—	—
	12^th^ August 2016^‡^	Bahawalpur	21.00	5	0.004	—	—	—	—	—	—
	21^st^ August 2016 ^‡^	Bahawalpur	12.33	5	0.030^†^	—	—	—	—	—	—
	17^th^ September 2016	Bahawalpur	0.37	5,10	0.860^NS^	1.00	5,60	0.403^NS^	0.58	25,60	0.866^NS^
*Trichoplusia orichalcea*	2^nd^ October 2015	Multan	2.17	5,10	0.139^NS^	6.64	3,36	0.005	1.33	15,36	0.267^NS^
	5^th^ January 2016	Bahawalpur	14.95	7,14	<0.001	17.16	4,64	<0.001	5.30	28,64	<0.001
	12^th^ August 2016	Bahawalpur	2.91	5,10	0.078^NS^	0.45	3,36	0.665^NS^	2.32	25,36	0.037^†^
	21^st^ August 2016	Bahawalpur	0.29	5,10	0.909^NS^	0.59	6,72	0.665^NS^	0.77	30,72	0.730
	17^th^ September 2016	Bahawalpur	0.22	5,10	0.947^NS^	0.20	3,36	0.872^NS^	1.16	15,36	0.351
*Plutella xylostella*	19^th^ January 2016	Multan	266.12	7,14	<0.001	456.16	9,144	<0.001	24.58	63,144	<0.001
	5^th^ January 2016	Bahawalpur	47.60	7,14	<0.001	262.75	4,64	<0.001	5.55	28,64	<0.001
	2^nd^ December 2016	Multan	108.60	5,10	<0.001	38.27	7,84	<0.001	12.86	35,84	<0.001
*Brevicoryne brassicae*	11^th^ December 2015^‡^	Bahawalpur	10.05	7	0.074^NS^	—	—	—	—	—	—
	5^th^ January 2016	Bahawalpur	38.41	7,14	<0.001	960.75	5,80	<0.001	5.08	35,80	<0.001
	2^nd^ December 2016^‡^	Multan	13.86	5	0.017^†^	—	—	—	—	—	—

^‡^Friedman’s test was used when the assumption of normally distributed residuals was not met despite log X + 1 transformation and removing sampling dates with zero insects present. Because Friedman’s test was performed on seasonal totals for assessing insecticide effects, the effect of sampling time and interactions between sampling time and insecticide, could not be assessed.

^†^Because several tests of the effects of insecticide, sample time and their interaction were carried out on each species we adjusted the significance criterion according to the Bonferroni procedure by dividing the significance criterion (0.05) by the number of times each species was evaluated. *P*-values that were no longer significant following adjustment are indicated with a dagger.

#### Brevicoryne brassicae

Spraying plots with imidacloprid every 7^th^ or 14^th^ day kept *B. brassicae* densities below 50 individuals per plant in trials intitiated on 11^th^ December 2015, while in control plots densities reached >1000 (Fig. 2a). Imidiacloprid was also effective in trials initiated on 2^nd^ December 2016 (Fig. 2c). In the 11^th^ December trial, NeemAzal was less effective than imidacloprid (Fig. 2a) but in the 2^nd^ December trial it was as effective as imidacloprid (Fig. 2c). In the trial initiated on 5^th^ January 2016, aphid densities were low (*ca*. <50 per plant) across all treatments including the control replicates (Fig. 2b). Neem seed extract suppressed aphid populations to lower than in control plots but not as greatly as did NeemAzal or imidacloprid (Fig. 2).

#### Plutella xylostella

Mean densities of <0.3 larvae per plant were frequently recorded in plots sprayed with Voliam Flexi every 5^th^ day (Fig. 3). Spraying Voliam Flexi every 10^th^ day suppressed the pest but less effectively than the more frequent application, and NeemAzal application gave similar results (Fig. 3). As for aphids, *P. xylostella* was suppressed by neem seed extract but not by as much as NeemAzal or Voliam Flexi (Fig. 3).

#### Spodoptera litura

In plots with Voliam Flexi sprayed every 5^th^ day, total larval densities were always <3 per plant throughout trial for trials initiated on 16^th^ July 2015, 12^th^ September 2015, 2^nd^ October 2015 and 17^th^ September 2016 (Supplementary Fig. S2a–c,f). For two remaining trials, inititiated on 12^th^ and 21^st^ of August 2016 at Bahawalpur, *S. litura* was the most numerous pest. However, considering total larval numbers did not facilitate the estimation of action threshold densities because pest abundance varied inconsitently across treatments (Supplementary Fig. S2d,e), chiefly due to high numbers of small larvae (Fig. 4a,d). We thus analysed *S. litura* numbers separately according to three larval size classes^36^: in all cases there were significant effects of insecticide treatment, sampling date and their interaction (Supplementary Table S2). Small larvae were highly abundant across all treatments in both trials (Fig. 4a,d) and action threshold densities were indeterminate. For medium sized and large larvae, application of Voliam Flexi every 5^th^ day led to mean weekly densities of <3 medium and <0.8 larve larvae per plant. Spraying Voliam Flexi every 10^th^ day or NeemAzal every 7^th^ day was in most instances as effective as Voliam Flexi every 5^th^ day (Fig. 4b,c,e,f). However, spraying with Voliam Flexi every 15^th^ day or neem seed extract was not generally as effective as other insecticides (Fig. 4b,c,e,f).

### Insecticides, *S. litura* egg batches and larval size

While *S. litura* larvae were observed in six of the ten trials (Supplementary Fig. S2), egg batches were only observed in four of these (Supplementary Fig. S3). The seasonal totals of egg batches observed (mean per plant weekly estimates, summed per treatment) were significantly affected by insecticide treatment for three of the four trials (16^th^ July 2015: *F*_5,12_ = 0.93, *P* = 0.492; 12^th^ August 2016: *F*_5,12_ = 9.62; *P* = 0.001; 21^st^ August 2016: *F*_5,12_ = 11.37; *P* < 0.001; 17^th^ September 2016; *F*_5,12_ = 20.91; *P* < 0.001; Supplementary Fig. S3). When egg batch numbers differed significantly across treatments, the highest numbers were observed in plots sprayed with Voliam Flexi every 5^th^ day (Supplementary Fig. S3b–d). However, there were significantly fewer egg batches in plots sprayed weekly with NeemAzal or neem seed extract than when sprayed with Voliam Flexi every 5^th^ day (Supplementary Fig. S3b–d). The numbers of egg batches observed were positively correlated with the numbers of small larvae (mean per replicate) present, uncorrelated with the number of medium sized larvae and either uncorrelated or negatively correlated with the numbers of large larvae present (Supplementary Table S3).

### Weight, marketability and revenue

Weight, marketability and revenue were affected by insecticide treatment in almost every trial (Supplementary Table S4). In general, control plots, with no insecticide applied, produced lightweight cauliflower curds (Supplementary Figs S4a and S5a) and the lowest proportions of marketable produce (Supplementary Figs S4b and S5b). In consequence, these also earned the least revenue (Supplementary Figs S4c and S5c). Plots in which synthetic insecticides or NeemAzal were applied produced heavier curds, a high proportion of marketable crop and earned high revenue (Supplementary Figs S4 and S5). The proportion of produce that was marketable was always greater than 90% when Voliam Flexi was applied every 5^th^ or 10^th^ day, and almost always when NeemAzal was applied every 7^th^ day (Supplementary Figs S4b and S5b). Spraying with neem seed extract achived this marketability criterion only when pest densities were low (both in control plots and neem seed extract treated plots). The revenue obtained was always highest in Voliam Flexi treatments; this was particulary so in trials in which pests were abundant overall and marketability was greatly affected. Plots sprayed with Voliam Flexi every 5^th^ day did not always result in higher revenue than plots sprayed every 10^th^ day, due to the higher pest control costs of the more frequent spraying (Supplementary Figs S4c and S5c). Spraying Voliam Flexi every 10^th^ day always gave higher revenue than weekly treatment with NeemAzal (Supplementary Figs S4c and S5c), due to the higher cost of NeemAzal.

Curd weight and the percentage of produce that was marketable were not affected by the abundance of small *S. litura* larvae but the correlation was highly negative and significant for medium and large larvae (Table 4), due to the higher rate of consumption by larger *Spodoptera* larvae^37,38^.

**Table 4:** Pearson correlation between *S. litura* larvae sizes and average weight or percent marketability.

Larvae sizes	Weight (g)	Marketability (%)
<1 cm	−0.174	−0.252
1–2 cm	−0.712***	−0.769***
>2 cm	−0.712***	−0.746***

Data used were from 12^th^ August, 21^st^ August and 17^th^ September trials conducted at Bahawalpur during 2016. *** indicates significance at *P* < 0.001.

### Action thresholds

Action thresholds were derived using peak pest density per plant observed across all sampling dates. As infestations of *B. brassicae*, *P. xylostella* and *S. litura* occurred across multiple sampling dates, we first determined that peak densities were correlated to ‘cumulative insect days’, which summarize the magnitude and duration of pest infestations^39,40^. Using weekly records per plant for *B. brassicae*, *P. xylostella* and *S. litura*^7,40^ we calculated the average number of each pest per plant from the current and previous evaluation dates and multiplied that by the number of days between the evaluations. These ‘insect days’ were then summed to provide ‘cumulative insect days’^39^. Regression analysis found strong correlations between peak infestation and cumulative insect days for *B. brassicae* (*F*_1,7_ = 3213.33; *P* < 0.001; *r*^2^ = 0.99), *P. xylostella* (*F*_1,7_ = 94.47; *P* < 0.001; *r*^2^ = 0.96) and *S. litura* (*F*_1,16_ = 143.54; *P* < 0.001; *r*^2^ = 0.90) (Supplementary Fig. S6) thus confirming that peak infestation is a candidate predictor for yield and is suitable for identifying action thresholds, as has been done in prior studies^7,41^. Action thresholds were then derived by identifying the peak pest density per plant from insecticide treatments that were able to attain >90% marketable yield. Treatments that could not attain high yield were considered ineffective both in terms of protecting yield losses and for deriving action thresholds.

*Brevicoryne brassicae* densities of up to 1000 individuals per plant were observed in unsprayed plots whereas spraying imidacloprid every 7^th^ day supressed aphid numbers to <50 per plant and the resulting crops had >90% marketability. However, application of imidacloprid every 14^th^ day, when overall aphid densities were high, did not supress aphid numbers sufficiently to achive 90% marketability. This was due to the longer time between consecutive sprayings allowing feeding to damage the crop. Applications of NeemAzal had inconsistent effects against aphids; in two trials suppressing densities below 50 per plant but in one trial densities remained around 100 per plant. Marketability of >90% was only attained when pest densities remained below 50 per plant. Thus, our recommended action threshold for insecticide application against *B. brassicae* is 50 individuals per plant.

*Plutella xylostella* mean densities were supressed better (<0.30 larvae per plant) in plots sprayed with Voliam Flexi every 5^th^ day than in plots (<0.55 individuals per plant) sprayed weekly with NeemAzal. However, both treatments always led to >90% produce being marketable. Thus, the action threshold density derived from spraying Voliam Flexi every 5^th^ day is 0.30 individuals per plant while it is 0.55 larvae per plant for NeemAzal application.

For *S. litura*, the action threshold densities based on the numbers of medium sized larvae were 3 per plant, derived from spraying Voliam Flexi every 5^th^ or 10^th^ day and NeemAzal on weekly intervals. The action threshold density for large larvae, which have high consumption rates^37,38^ and tend to migrate from leaves to curds (Supplementary Fig. S7b–g), was also 3 per plant for NeemAzal, and <0.80 per plant for Voliam Flexi every 5^th^ day.

## Discussion

While straightforward strategies of applying insecticides at pre-determined times may be preferred, over integration of multiple control methods, by growers due to their operational simplicity and perceived effectivity^42^, such approaches are likely to fail in the absence of action threshold guidelines^43^. Action threshold based guidelines have been developed for cruciferous pests^44^ but thresholds can vary according to pest species (e.g. due to differing consumption rates)^41,45^ and may also differ regionally^46^. Here we considered a range of pest species as well as seasonal variation to obtain a broad overview of pest activity periods, persistence and abundance. This information enabled us to identify major and minor cauliflower pests and the action thresholds for the major pests. In other crops, action thresholds have been developed using degree day models^47^ or using pheromone trap catches^48^. They can also be established using damage-based criteria^11^, the percentage of infested plants^41^ or counts of pest densities^7^. The most commonly adopted approach is comparison of a set of potential action thresholds and their subsequent yield responses^7,41^. As there are few, if any, prior evaluations of action thresholds for most cauliflower pests, there was little information on the pest densities that would be relevant to evaluate. We therefore applied insecticides at predetermined intervals to obtain a range of pest infestations and thus establish relationships between pest infestation and marketable yield, allowing us to identify action thresholds based on pest count criteria. This enabled us not only to identify action thresholds but also the most promising types of insecticidal treatments.

Cauliflower crops were attacked by several species of lepidopterans at low density for short periods in only the first year of our trials: *Helicoverpa armigera* and *Trichoplusia orichalcea* have not been reported previously as severe pests of cauliflower whereas *Spodoptera exigua* has commonly been reported as serious pest of cabbage in other countries^49,50^ but not in Pakistan. As these species have the potential to cause economic damage in other crops, we suggest that cauliflower growers monitor their presence but are unlikely to need to spray against these pests. These minor pests are also likely to be controlled as an indirect consequence of insecticide applications triggered by the presence of locally major pests^36^.

We identified three species, all of which are known pests of brassicas^25,31^, to be major pests: the aphid *B. brassicae* and the lepidopterans *P. xylostella* and *S. litura*. All three occurred in numbers sufficient to cause economic damage but, as found in previous studies^14,51,52^, their numbers varied greatly within growing seasons and were affected by insecticide treatment. Cauliflowers sown between July and October are at risk of *S. litura* infestation with the loss of marketable produce, if left unsprayed, ranging between 42–62% for crops sown in July or August and between 12–44% for crops sown in September to October (different cultivars were used at different times of the year). The extent of crop losses was greatest when the sowing time coincided with the initial appearance of the pest, with *S. litura* reaching peak abundance in mid-September. Crops sown from December to January are at risk of infestation by *B. brassicae* and *P. xylostella*. These pests either appeared alone or concurrently, and losses in unsprayed plots were higher in December (63–84%) than in January sowings (21–26%). As for *S. litura*, crops sown at times coinciding with the initial appearance of *P. xylostella* and *B. brassicae* were the most vulnerable to damage. As market price varied within planting dates across seasons, planting date adjustment is unlikely to be adopted by commercial growers but may be valuable for subsistence growers. However, information generated through inclusion of planting dates in this study provides useful information to both commercial and subsistence growers on pest activity periods, infestation rates and thus the intensity of control required.

For *B. brassicae* >90% marketability could be attained when pest densities remained below 50 per plant, which is our recommended action threshold for insecticide application against this pest. As the seedling and pre-cupping stages of the crop are more prone to infestation^53^, it will be especially important to monitor aphid densities during these periods. We also note that plants may appear to be uninfested but on closer inspection may harbour large colonies, with the potential to expand rapidly, concealed between leaf folds (Supplementary Fig. S8); therefore it is recommeded to check carefully whole plants for the presence of aphids.

For *P. xylostella* the action threshold density is recommended as 0.30 individuals per plant for applying Voliam Flexi or 0.55 larvae per plant when applying NeemAzal. Other studies have found that action thresholds vary according to the insecticide used^6,54^. We also observed that spraying Voliam Flexi every 10^th^ or 15^th^ day was effective in reducing larval densities below 0.55 individuals per plant but, unlike NeemAzal, neither of these treatments could guarantee high marketability due to the long periods between treatments during which any larvae present were able to feed. Adopting NeemAzal can therefore reduce the intensity of pest control effort and yet achieve high marketability. *Plutella xylostella* can feed on leaves, on the whorl of leaves surrounding cauliflower or on the curd itself (Supplementary Fig. S9); therefore their presence should be carefully monitored across the whole plant.

*Spodoptera litura* was present in six of the ten trials and its abundance was affected by insecticide treatment in three of these. This may have been due to variation in planting densities associated with the use of different cultivars: the three trials in which insecticide treatment affected *S. litura* abundance were all Sathra cultivar cauliflowers planted on one side of the bed and thus the spray could easily reach all sides of each plant better than double-planted cultivars. Further, host plant identity can influence insect susceptibility to insecticides^55^: as a response to herbivore feeding, plant-produced allelochemicals enhance release of metabolizing enzymes that might also enhance detoxification of insecticide active ingredients^55^.

When we considered the total numbers of pest larvae (2015 trials), we were able to derive action threshold densities when abundance was low. In these cases, the action threshold was <3 larvae per plant, derived from the application of Voliam Flexi every 5^th^ or 10^th^ day and from NeemAzal at weekly intervals. Total larval numbers are high following oviposition and eggs are typically laid in batches of several hundred^56^ (Supplementary Fig. S7a). We were unable to derive action threshold densities when abundance was high because none of the insecticide treatments suppressed pest densities below 3 larvae per plant. In trials carried out in 2016, we considered the size classes of *S. litura* larvae separately. Small larvae had no discernible effect on crop weight and marketability, and are thus unimportant for deriving action thresholds. For medium sized larvae *S. litura*, the AT was 3 per plant, derived from spraying Voliam Flexi every 5^th^ or 10^th^ day and from weekly application of NeemAzal. For large larvae, ATs were 3 per plant, derived from spraying NeemAzal at weekly intervals, and <0.80, derived from spraying Voliam Flexi every 5^th^ day. Previous studies developed fixed schedules for decision making against *S. litura*^31^ but our findings suggest that the ability of *S. litura* to cause damage varies according to larval size class. Therefore, growers should use information on both the numbers and the developmental stages of the pest (phenology) in their pest management decisions.

Adult *S. litura* are likely to prefer laying eggs on healthy, competitor-free plants^57^ and *S. littoralis* females avoid oviposition on damaged cotton, *Gossypium hirsutum*, which may be mediated by herbivore-induced plant volatiles. If an insecticide does not act as an ovipositional deterrent, the probability of eggs being laid on sprayed plants may be higher than on untreated plants. Chlorantraniliprole typically acts via high toxicity to neonates emerging from eggs^58^. However, its mode of action can induce sub-lethal effects, such as reduced reproductive potential^59^. In contrast, neem-derived formulations act both on oviposition behaviour and as insecticides^29,60^; the numbers of egg batches observed in plots treated with NeemAzal or neem seed extract were consequently low. As both Voliam Flexi and NeemAzal are lethal to newly hatched larvae^58,61^, there were many small (early instar) larvae present in some plots between spraying dates but medium or larger sized larvae were rare.

Overall, synthetic insecticides were effective in reducing pest densities and improving cauliflower crop yield in our trials. Trailing the botanically derived commercial formulation NeemAzal indicated that it is as effective as the synthetic insecticides in terms of pest suppression and production of marketable yield. NeemAzal application cost almost three times as much as the synthetic insecticides and thus netted less revenue, despite its equal effectiveness in protection against detrimental effects on marketability of individual curds. Our self-prepared neem seed extract was substantially less expensive but also less effective in terms of suppressing lepidopteran and aphid pest numbers but, along with NeemAzal, was very effective in terms of reducing the number of *S. litura* egg batches laid, in accord with a prior report on the effectivity of self-prepared neem extract^29^. Neem-derived compounds may operate via effects on multiple life-history and behavioural parameters: deterring oviposition, as we found for *S. litura* when using both commercial and self-prepared neem formulations, disrupting development and by inhibiting feeding, as with other pest species^62–64^. These compounds may keep pests under physiological stress, facilitating susceptibility to natural enemies^65^ and this may also lead to action thresholds being higher than for synthetic insecticides, thus reducing the overall intensity of application needed. In our trials, plots treated with neem seed extract always had better yields than untreated control plots. These properties, and the potential for use in pesticide resistance management strategies^66^, favour their consideration for cautious adoption into IPM programmes^31^.

In conclusion, unless managed, *B. brassicae*, *P. xylostella* and *S. litura* caused substantial reductions in marketable yield. Neem-derived alternative insecticides were as effecitve as synthetics in managing cauliflower pests and in protecting yield. NeemAzal deterred *S. litura* ovipositon better than Voliam Flexi, and also NeemAzal-derived ATs for informing pest management against *S. litura* and *P. xylostella* were higher. Botanicals present a multitude of chemisteries for developing pest mangement products and their use is increasing, especially in developing countries. Given that they also can have undesired effects in agro-ecosystems; their inclusion into pest control programs should be cautious and involve the use of ATs. Our work indicates that cauliflower crops can yield high marketability if the per plant densities are below 3 medium-sized larvae for *S. litura*, 0.3 to 0.55 larvae for *P. xylostella* and 50 individuals for *B. brassicae*. Therefore, these threshold densities can be used as decision support tools for triggering the application of insecticide. The implementation of the AT-based approach involves regular pest monitering, can reduce pesticide use^67^ and increase revenue compared to fixed spray schedules^46^; nontheless, field experiments that formally evaluate the performance of the ATs derived in this study against fixed-scheduled spraying have yet to be carried out. Further, ATs can vary regionally, depending upon the composition of pest species that are present and their consumption rates. The relatively simple approach we have used for deriving ATs can be applied to other regions and crops and the marketability criterion can be adjusted according to standards accepted by commercial or subsistance growers. As the continued use of any given insecticide is likely to select for resistance, further studies should consider developing long-term strategies that involve the application of several formulations, with low non-target and polluting effects, alongside employing the action threshold approach to reduce the total amount of pesticide applied.

## Methods

### Insecticides

Voliam Flexi (VF; a mix of chlorentraniliprole and thiamethoxam) was obtained from Syngenta Crop Science, Karachi, Pakistan. Chlorentraniliprole, an anthranilic diamide insecticide, acts by selectively binding to ryanodine receptors in muscle cells, resulting in the uncontrolled release of calcium stores^68^, is typically used against lepidopterans. Thiamethoxam, a neonicotinoid, acts selectively on the insect nicotinic acetylcholine receptor^69^. Imidacloprid (I) another neonicotinoid, registered under the trade name “Confidor”, was obtained from Bayer Crop Science, Karachi, Pakistan, is mostly used against sucking pests including aphids^30^. The use of some diamides has been restricted in the USA^70^ and the use of neonicotinoids has been restricted by the European Union and United Kingdom since 2013 but they are still being applied in many of the world’s cropping systems^30,71^.

The botanically derived NeemAzal (NA; azadirachtin-A (10 g/L)), was obtained from Trifolio GmbH, Germany. NeemAzal was first registered in Germany in 1998 as plant protection product and, along with other neem-derived biopesticides, is registerd in many other parts of the world^72,73^. Neem seed extract (NSE) was prepared following methods given in Boursier, *et al*.^27^: briefly, about 100 grams depulped seeds were ground in an electric blender. The resulting powder was tied in a muslin cloth, and soaked for 7 days in 1 L of water, yielding aqueous extract.

Field recommended doses of Voliam Flexi (51.96 g/ha) and imidacloprid (98.9 ml/ha) were mixed in one liter of water at rate of 0.17 g and 0.33 ml, respectively, for spraying. NeemAzal was mixed in water (1.2 ml/L) before application. NSE was further diluted to 5% in water (50 ml/L) before application. All insecticides were applied as foliar sprays using a hand operated knapsack sprayer (PB-20; Cross Mark Sprayers, Johor, West Malaysia) fitted with a hollow cone nozzle. Separate sprayer tanks were used for botanical and synthetic insecticides. Between 5 and 7 liters was sufficient to spray the replicates of each of the treatments, with the exact amount depending on crop stage and planting density.

### Field trials

Field experiments were conducted during the cauliflower growing seasons of 2015–16 and 2016–2017 in the Southern Punjab, Pakistan. Overall, there were ten experimental plantings (‘trials’) of cauliflowers belonging to six season-specific cultivars (Table 1). Cultivars were chosen on the basis of a history of good general performance at a given time of year and were the varieties favoured by local farmers. Six experiments were conducted at Moza Bindra, Bahawalpur (29°41′93.2″N, 71°64′73.4″E), three at Moza Kayaanpur, Multan (30°12′78.0″N, 71°45'58.5″E) and one trial was carried out at the research farm of Bahauddin Zakariya University (BZU), Multan (30°25′70.5″N, 71°51′22.1″E). Two further crops were planted in Multan on 3^rd^ July 2016 and 3^rd^ January 2017 but could not continue due to no pest present in the first and flooding of seedlings in the second.

Experimental cauliflowers were mostly raised by using nursery prepared 4–5 week old seedlings whereas sometimes seeds were sown directly into beds by manual dibbling (3–4 seeds per dibble and thinned to one plant following germination) (Table 1). Single side beds (100 cm apart) were chosen for July-August sowings where it was necessary to pile up the soil from other side of the bed to support large plants (Table 1). Double side beds (45 cm apart) were chosen for September-January sowings and earthing-up was not needed. Treatment plots consisted of six single-planted beds or four double-planted beds; length of the bed was 6 m in both cases. Seedlings were spaced 30 cm apart along the rows. The method of sowing followed local grower practice.

Initially, plantings were divided into three equal blocks and visited twice per week to monitor pest presence. Once pests were observed, treatment plots (three replicates each) were identified within blocks, following a randomized complete block design, and insecticide spraying commenced. There were six main insecticidal treatments used against lepidopterans: (1) no insecticidal application (control), (2) Voliam Flexi (VF) application every 5^th^ day, which is representative of typical application by cauliflower producers in Pakistan, (3) VF application every 10^th^ day, (4) VF application every 15^th^ day, (5) NeemAzal (NA) application every 7^th^ day and (6) Neem seed extract (NSE) application every 7^th^ day. Because December to January sown cauliflower crops became infested with aphids, we included imidacloprid in these trials (Table 1): this is also a typical application procedure by cauliflower producers in Pakistan. In three trials (1^st^ December 2015, 5^th^ January and 19^th^ January 2016), we also tested fortnightly applications of botanicals (NSE and NeemAzal) but these proved less effective than their weekly-sprayed counterparts (see below) and were therefore not considered in subsequent trials (Table 1).

Pest sampling was carried out at weekly intervals until harvest. At each visit, ten plants from each plot (30 plants per treatment) were selected randomly and aphids, lepidopteran larvae and *S. litura* egg batches were counted. In 2015–16 trials, individual *Spodoptera* larvae were counted irrespective of their size whereas following year, they were counted by size class (small <1 cm, medium 1–2 cm and large >2 cm in length)^38,74^. Voucher specimens of these pests were deposited in the IPM laboratory at BZU, Multan, Pakistan. Aphids and lepidopteran pests were identified on the basis of morphology^75–78^.

Harvesting was initiated when 80–90% cauliflowers attained marketable size. One hundred cauliflower curds (the edible white portion) from randomly selected plants were harvested per treatment. Their circumference was measured using tape and curd diameter was calculated as equal to circumference/π^79^. Curds with diameter <10 cm or those showing insect feeding scars or which were contaminated with frass, were deemed unmarketable^46^. Local growers attempt to achieve >90% marketable yield, thus we used this as the criterion for deriving action thresholds.

Financial revenue was calculated following the method of Stewart and Sears^46^, which considers pesticide purchase and application costs, crop yield and crop value: revenue is the value of the yielded crop minus pest control costs. Pest control costs were the total spent on purchase of an insecticide and on its application. Purchase costs (in US dollars) were $19.76/ha for Voliam Flexi, $17.29/ha for imidacloprid, $6/ha for neem seed extract and $64.43/ha for NeemAzal. The application cost for each treatment was $6.92 per hectare. Marketability data for each insecticide treatment were converted to yield/ha, which was used for estimation of market value of the crop. Marketable produce was sold at prices reflecting local market conditions (Supplementary Fig. S10).

### Statistical analysis

Analyses were performed using the software package *GenStat* (version 17, VSN International, Hemel Hempstead, UK).

#### Seasonal pest totals

Weekly records of each pest species were pooled to provide seasonal totals, which were used for assessing insecticides and planting date effects on the abundance of each pest species or their composition (across species). These effects on pest composition were examined by multivariate analysis of variance (MANOVA) using data from all trials conducted in a particular site in that particular year. Note that because the cauliflower cultivars used varied during each season (Table 1) this confounds with sowing date variation: we report results in reference to sowing date for simplicity. For individual pest species, these effects were assessed using analysis of covariance (ANCOVA), with insecticide treatment fitted as a factor and sowing date as a covariate. Because several ANCOVA tests were carried within each year and each site, we assessed significance following Bonferroni correction, dividing the standard significance criterion (*P* < 0.05) by the number of tests (=number of species present) at each site in each year. Effects of insecticide treatments on total counts of *S. litura* egg batches per plant were assessed using ANOVA. Effects of *S. litura* batches counts on the abundance of small, medium and large sized larvae were assessed using Pearson correlations.

#### Within-season pest abundance

For each trial, the impact of insecticides on weekly abundance of each pest species present was assessed using repeated measures ANOVA, with insecticides and sampling dates treated as factors. Due to the repeated sampling within each trial, the degrees of freedom (*df*) were adjusted by Greenhouse-Geisser epsilon correction factors. Count data were +1 log_10_ transformed to improve compliance with the assumptions of normality and homogeneity of variances. When these assumptions were not met, as assessed by residuals plots, this was typically due to no insects being present on some sampling dates. In these instances, we re-analysed by excluding data from dates with zero insects present. Following this, there were cases in which the assumption of homogeneous variances was not met. As this can generate Type 1 errors, we re-tested for insecticide effects using non-parametric Friedman’s tests on seasonal total numbers of pest species present. Because several tests of the effects of insecticide, sample time and their interaction were carried out on each species, we employed the Bonferroni correction, dividing the standard significance criterion (*P* < 0.05) by the number of times each species was evaluated.

#### Weight, marketability and revenue

Treatment effects on curd weight, percent marketability and revenue were assessed using ANOVA. Percent marketability data were arcsine-square root transformed before analysis. Effect of *S. litura* larvae sizes on curd weight and percent marketability were assessed using Pearson correlations.

### Ethical Approval (Research involving human participants and/or animals)

No specific permits were required for the experiments conducted.

## Supplementary information

Supplementary Information

## Data Availability

The datasets generated and analysed during this study are available from the corresponding authors on reasonable request.
