# Supplementary material for: Field evaluation of synthetic and neem-derived alternative insecticides in developing action thresholds against cauliflower pests
Source: Sci Rep. 2019 May 22;9:7684. doi: 10.1038/s41598-019-44080-y (PMC6531477; doi:10.1038/s41598-019-44080-y)
Supplement: Supplementary file 1 — Supplementary Information [file 41598_2019_44080_MOESM1_ESM.pdf]

Supplementary materials for:

Field evaluation of synthetic and neem-derived alternative insecticides in developing action thresholds against cauliflower pests

Farhan Mahmood Shah[1], Muhammad Razaq[1*], Qasim Ali[1,2], Sarfraz Ali Shad[1], Muhammad Aslam[3] and Ian C.W. Hardy[4*]

[1] Department of Entomology, Faculty of Agricultural Sciences and Technology, Bahauddin Zakariya University, Multan 60000, Pakistan

[2] Faculty of Forestry and Wood Sciences, Czech University of Life Sciences, Kamýcká 1176, Czech Republic

[3] COMSATS Institute of Information Technology, Vehari, Pakistan

[4] School of Biosciences, University of Nottingham, Sutton Bonington Campus, Loughborough, UK

***Correspondence to:**

Dr Muhammad Razaq
Department of Entomology, Faculty of Agricultural Sciences and Technology, Bahauddin Zakariya University, Multan 60000, Pakistan
Tel: +9261 4507544
Email: muhammadrazaq@bzu.edu.pk

and

Dr Ian CW Hardy
School of Biosciences, University of Nottingham, Sutton Bonington Campus, Loughborough, LE12 5RD, UK
Tel: +44 115 95 16052
Email: ian.hardy@nottingham.ac.uk

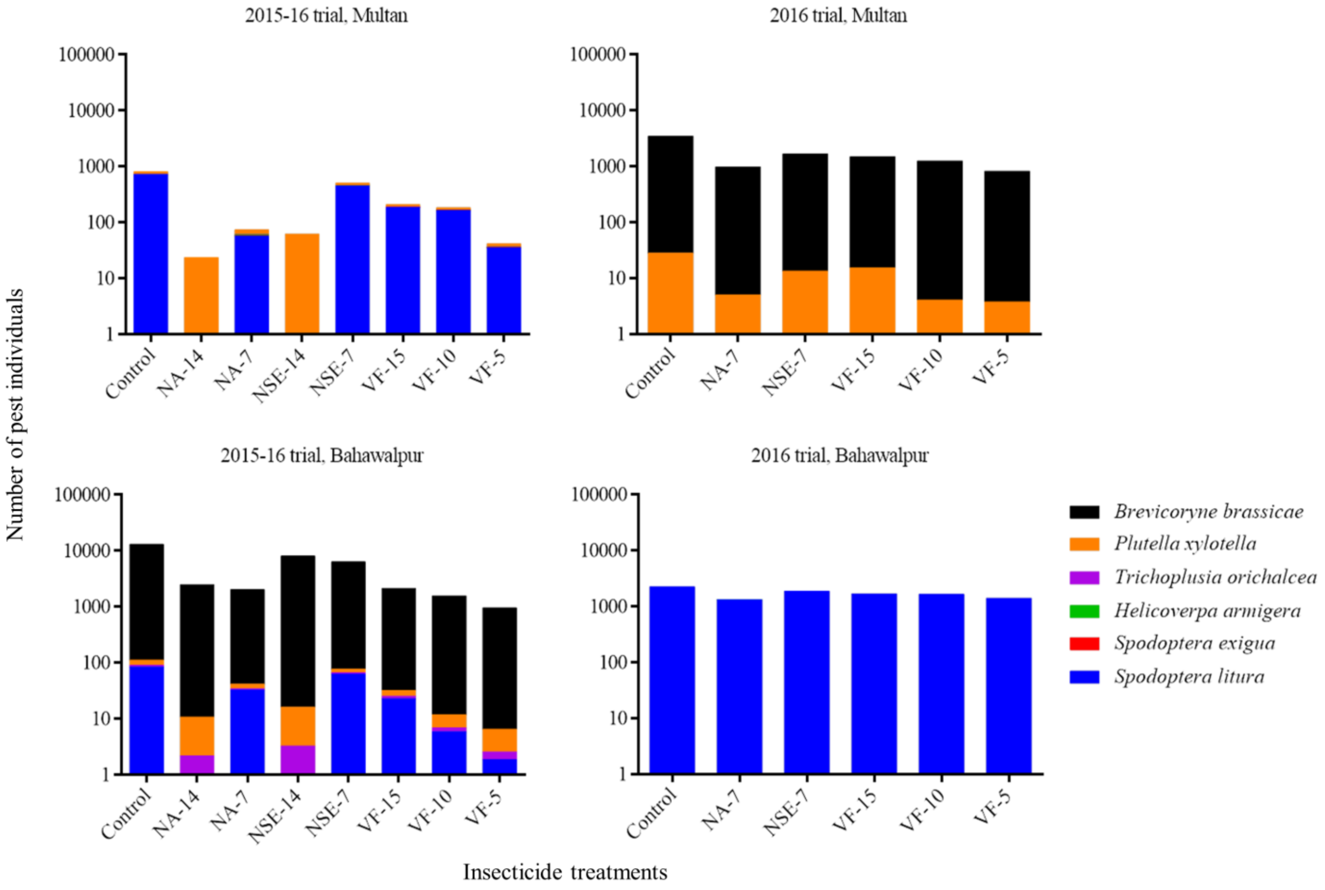

Supplementary Fig. S1 Seasonal profiles of pest species present. Each panel shows the totals for one site in one year. Bars are stacked. Control = no insecticidal application, NA-7 = NeemAzal application every 7th day, NSE-7 = Neem seed extract application every 7th day, VF-15 = Voliam Flexi application every 15th day, VF-10 = Voliam Flexi application every 10th day, VF-5 = Voliam Flexi application every 5th day. (Note log scale)

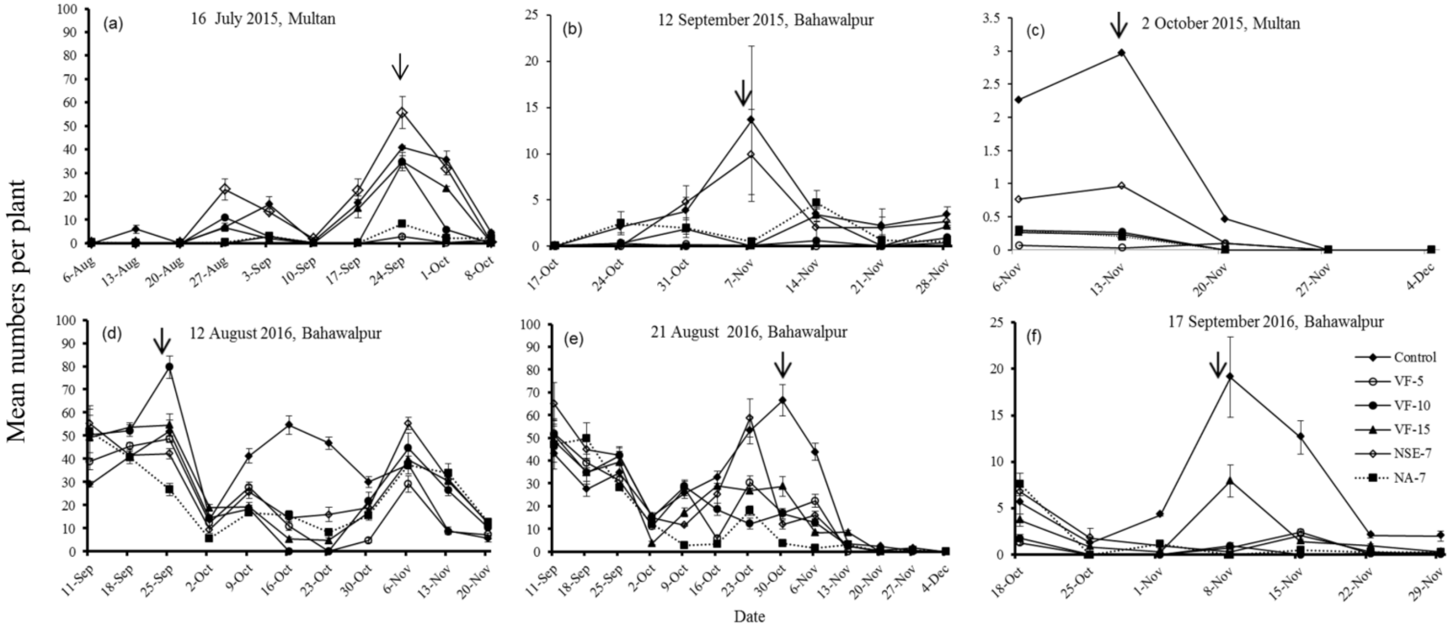

Supplementary Fig. S2 Effect of insecticide treatments on the weekly (mean ±SE) numbers of *S. litura* larvae following different planting dates. Control = no insecticidal application, VF-5 = Voliam Flexi application every 5th day, VF-10 = Voliam Flexi application every 10th day, VF-15 = Voliam Flexi application every 15th day, NA-7 = NeemAzal application every 7th day, NSE-7 = Neem seed extract application every 7th day. Arrows indicate dates when the pest population reached a peak in the untreated plots. (Note the differing y-axis scales)

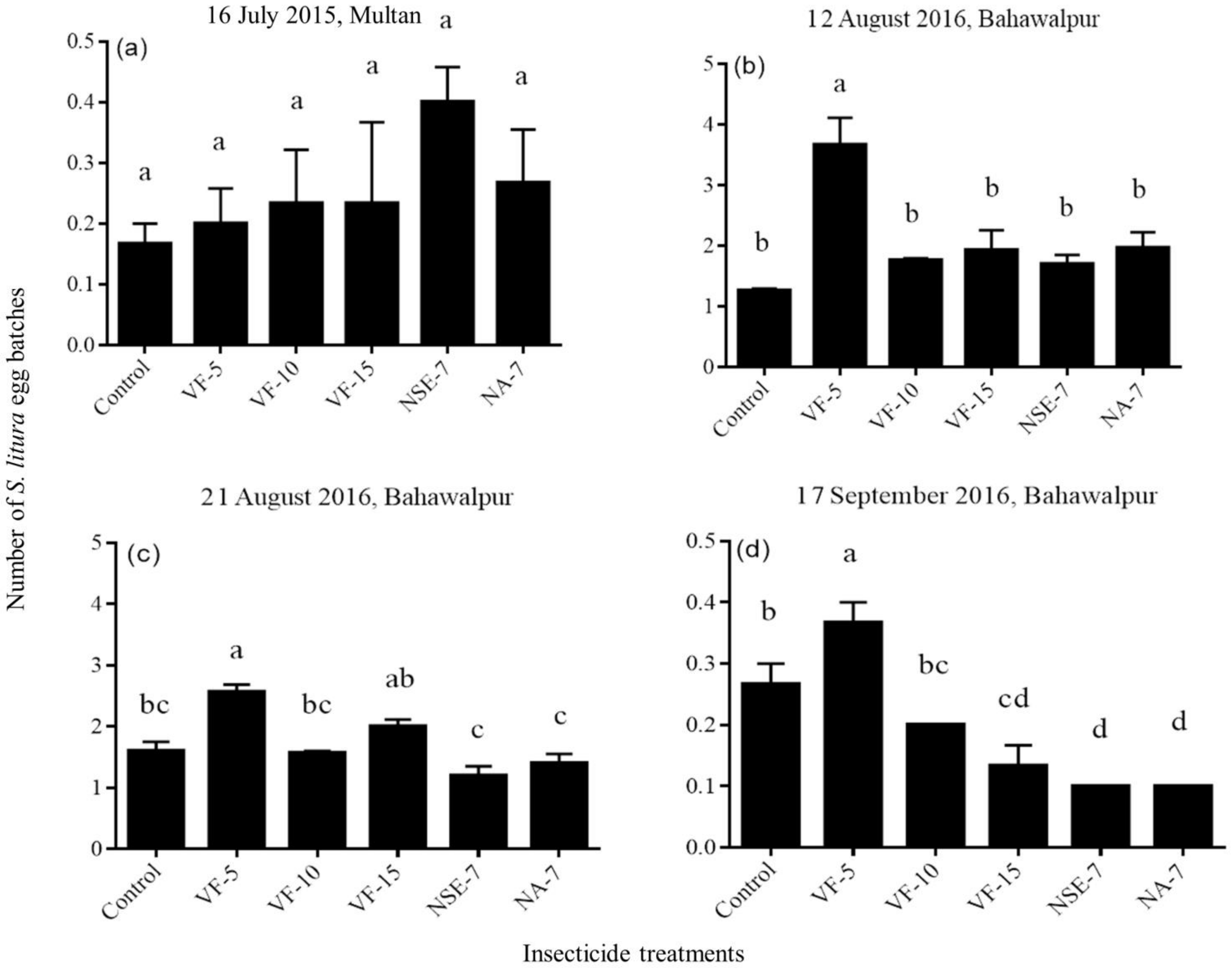

Supplementary Fig. S3 Effect of insecticide treatments on the seasonal totals of *S. litura* egg batches (mean per plant)**.** For the six trials, for which data were not shown, no egg batches were found. Insecticide treatments are as defined in Supplementary Fig. S2. Bars sharing common letters do not differ significantly (*P*>0.05; Tukey HSD test). (Note differing y-axis scales)

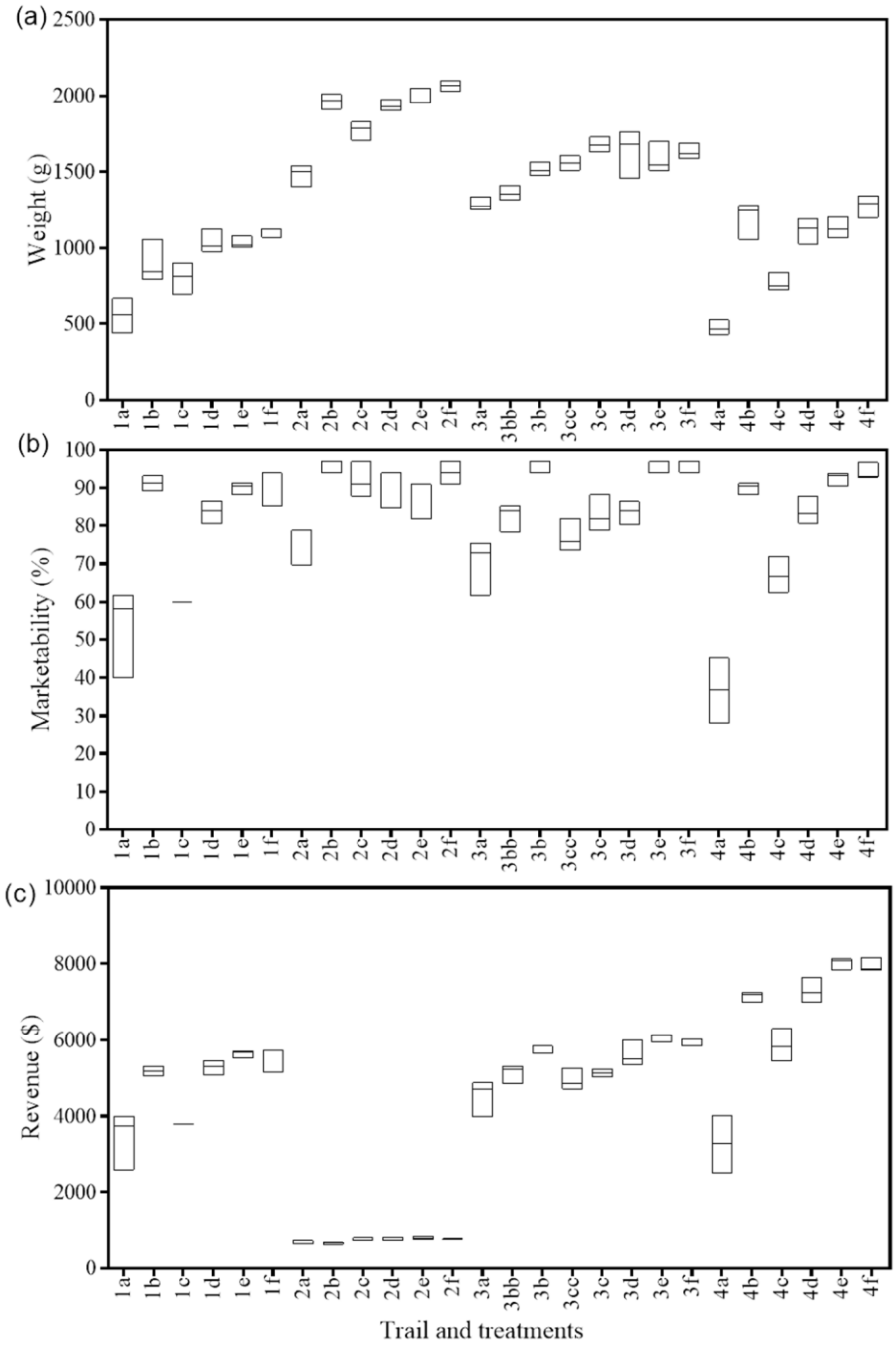

Supplementary Fig. S4 Cauliflower weight, marketability and revenue obtained from Multan trials. Bars within boxes indicate median values. On the x-axis, numbers indicate the sequence of trials (as in Table 1). Letters indicate treatments: a = control; b and bb, c and cc indicate NeemAzal and neem seed extract (NSE) treatments sprayed on weekly and biweekly intervals, respectively; d, e, and f indicate Voliam Flexi treatments sprayed on every 15th, 10th and 5th day, respectively

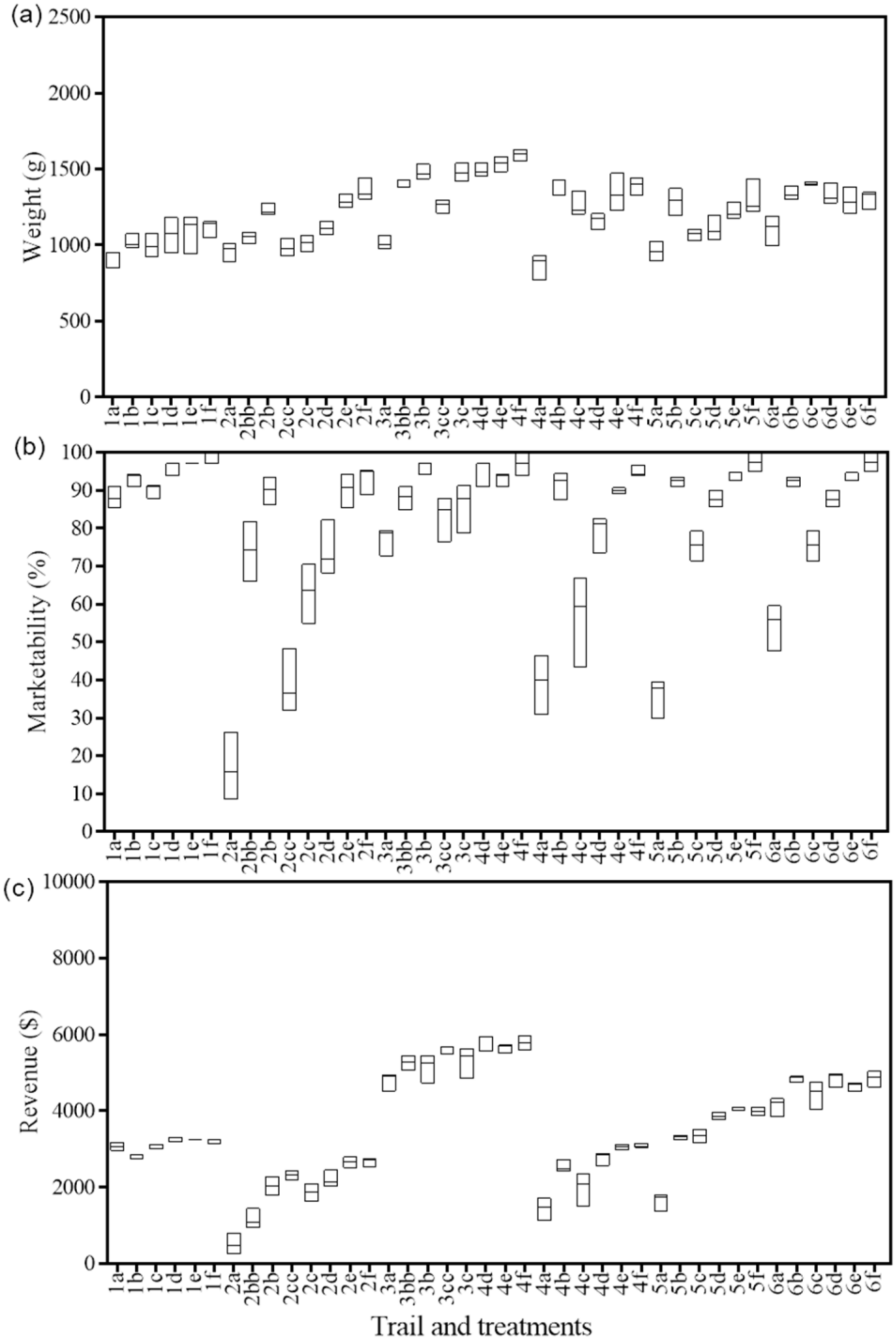

Supplementary Fig. S5 Cauliflower weight, marketability and revenue obtained from Bahawalpur trials. Bars within boxes indicate median values. On the x-axis, numbers indicate the sequence of trials (as in Table 1). Letters indicate treatments: a = control; b and bb, c and cc indicate NeemAzal and neem seed extract (NSE) treatments sprayed on weekly and biweekly intervals, respectively; d, e, and f indicate Voliam Flexi treatments sprayed on every 15th, 10th and 5th day respectively

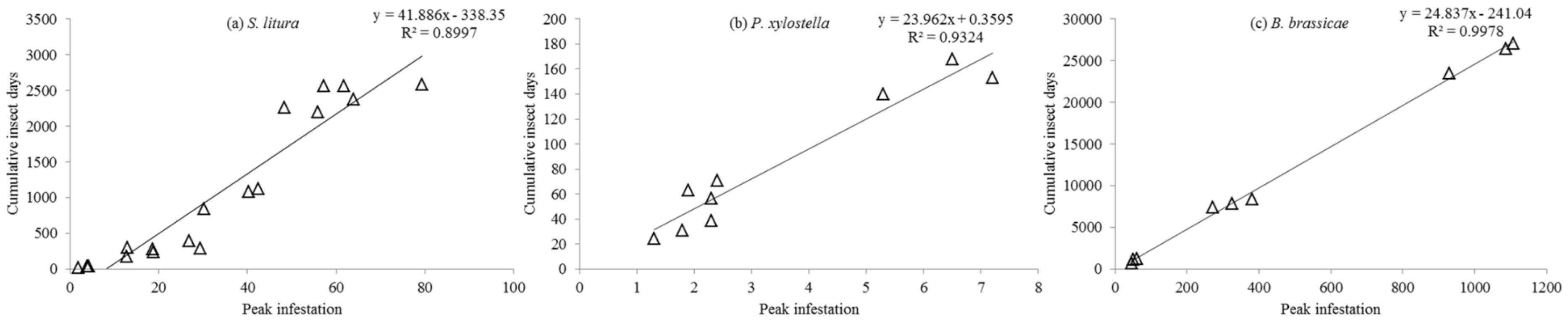

Supplementary Fig. S6. Relationship between peak infestation and cumulative insect days (data are mean per replicate, from untreated plots)

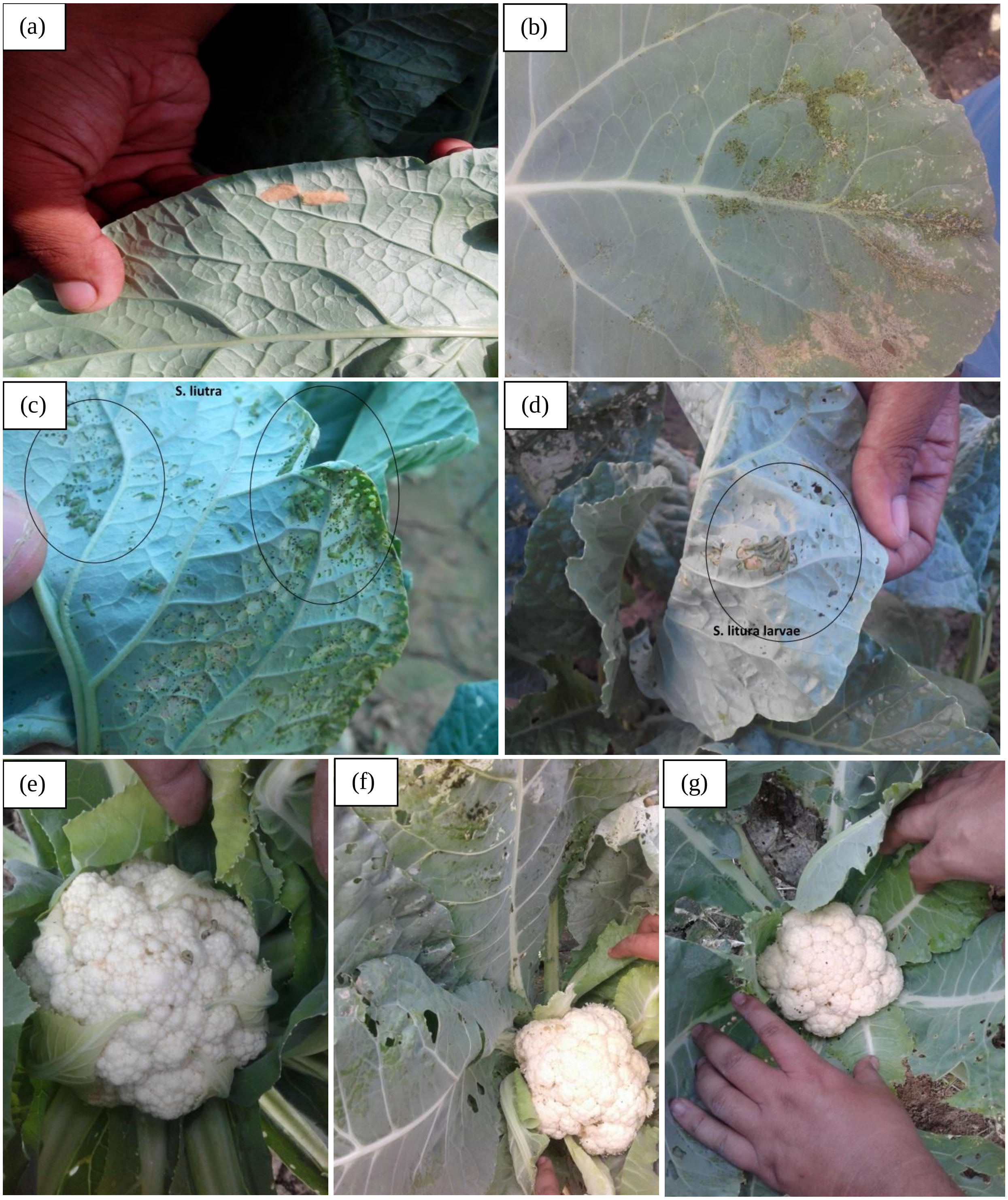

Supplementary Fig. S7. Presence of *Spodoptera litura* on cauliflower plants. (a) Egg batches on leaves, (b) small larvae on leaves, (c) medium sized larvae on leaves, (d) large larvae on leaves, (e-g) large larva on curds.

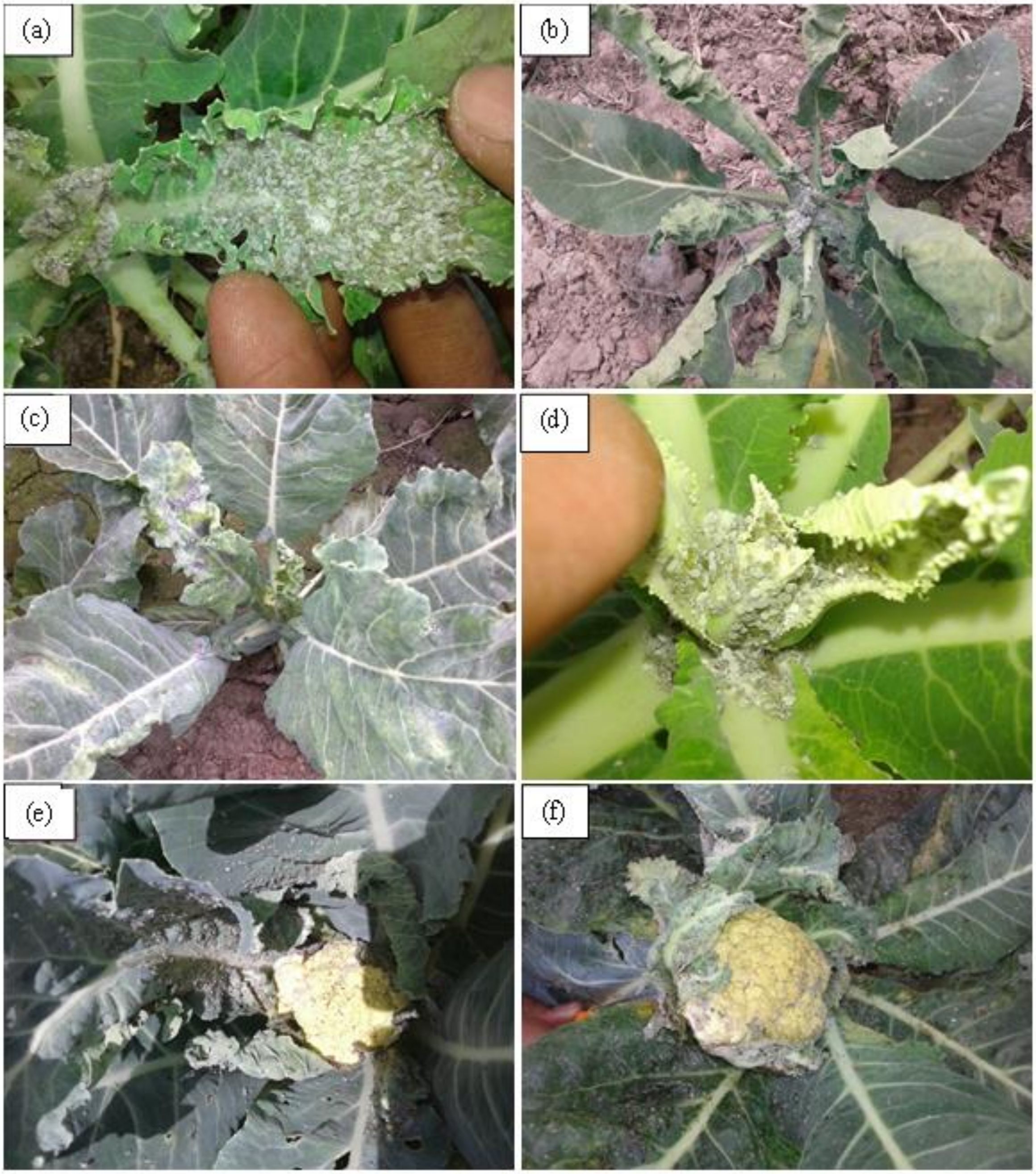

Supplementary Fig. S8. Presence of *Brevicoryne brassicae* on cauliflower plants at different growth stages. Aphid were present on leaves (a-c) and growing points (c-f)

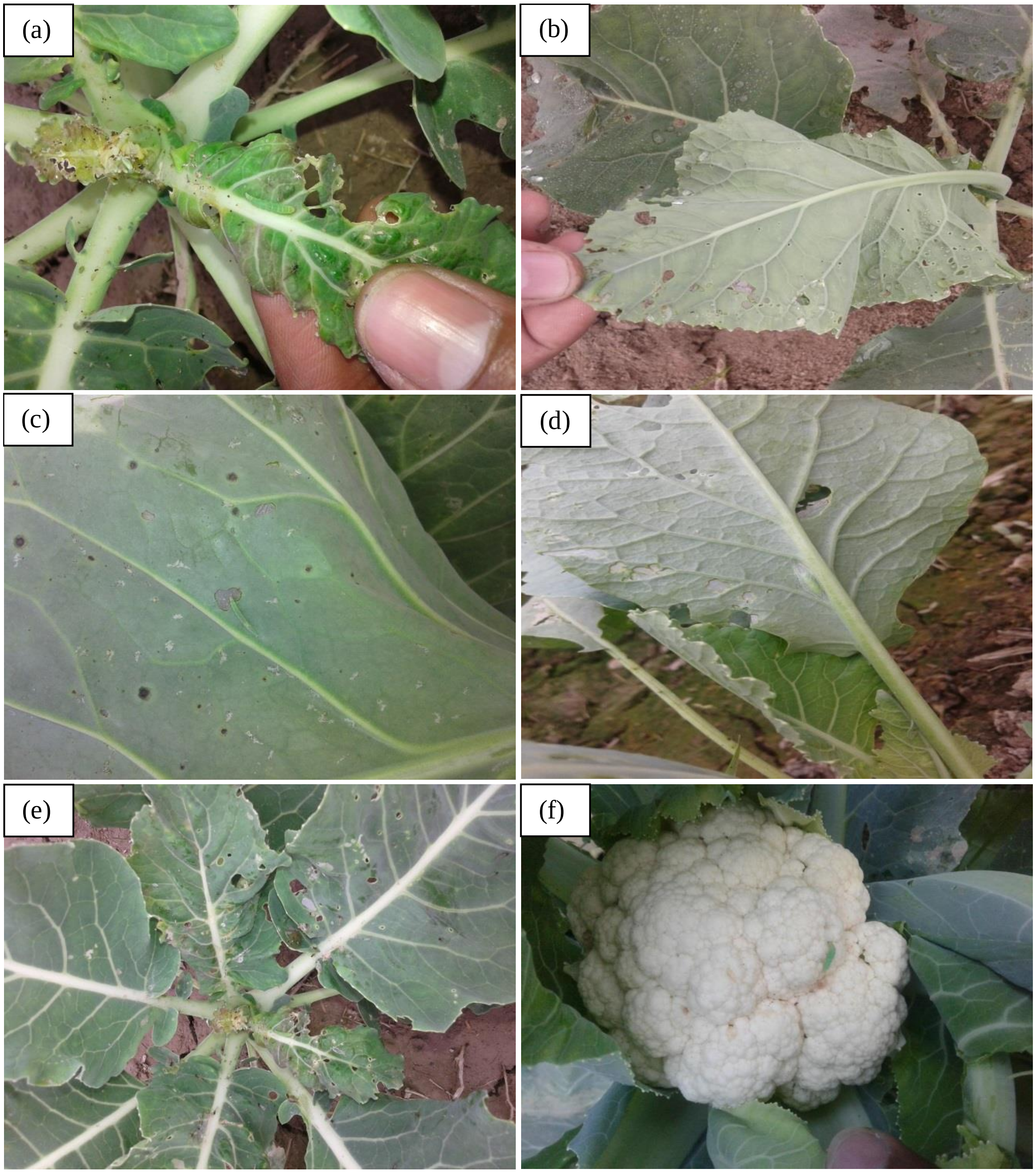

Supplementary Fig. S9. Presence of *Plutella xylostella* on cauliflower plants at different growth stages. Larvae feeding on leaves (a-e) and growing points (f)

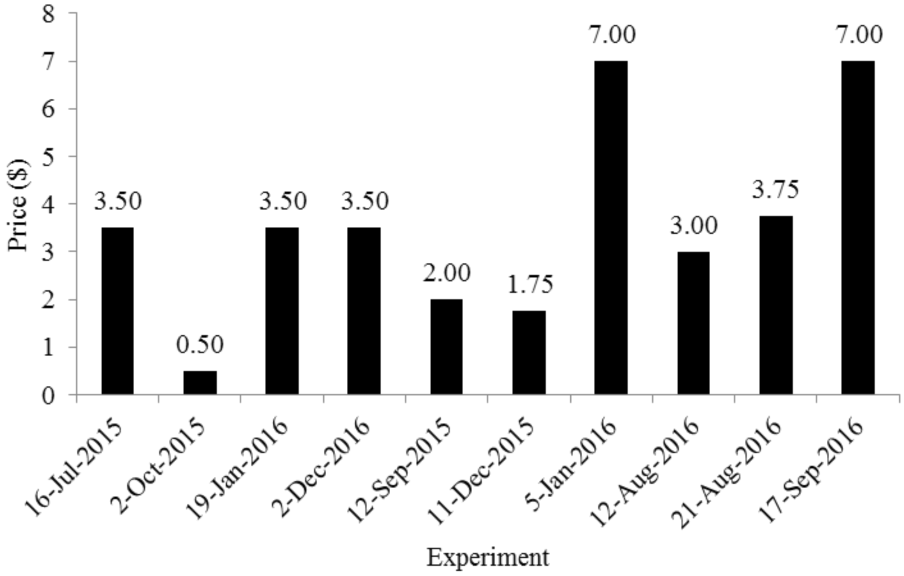

Supplementary Fig. S10. Sale price (in US dollars) of marketable produce (in bags) for trails according to local markets. One bag contained overall 13 cauliflower curds at Multan; and 27 cauliflower curds at Bahawalpur.

Supplementary Table S1. Seasonal totals of insect pest numbers per plant from all treatments combined

Pest species	Multan trials		Bahawalpur trials	
	2015-16	2016-17	2015-16	2016-17
Spodoptera litura	1629	-	214	10281
Spodoptera exigua	6	-	-	2
Plutella xylostella	269	70	77	-
Helicoverpa armigera	10	-	-	8
Trichoplusia orichalcea	3	-	22	3
Brevicoryne brassicae	-	9691	36559	-
Overall	1917	9761	36872	10294

Supplementary Table S2. Effects of insecticide treatments on mean weekly numbers of small, medium and large *S. litura* larvae in August planted cauliflowers during 2016 at Bahawalpur

Planting date	Size	Insecticide			Sample time			Insecticide × sample time interaction		
		F	*df*	*P*	*F*	*df*	*P*	*F*	*df*	*P*
12th August	<1cm	5.95	5,12	0.005	447.72	10,120	<0.001	69.06	50,120	<0.001
	1-2cm	147.48	5,12	<0.001	47.55	9,108	<0.001	14.01	45,108	<0.001
	>2cm‡	14.04	5	0.015	-	-	-	-	-	-
21st August	<1cm	66.32	5,12	<0.001	256.33	9,108	<0.001	22.74	45,108	<0.001
	1-2cm‡	14.61	5	0.012	-	-	-	-	-	-
	>2cm‡	14.61	5	0.012	-	-	-	-	-	-

‡ Friedman's test was used when the assumption of normally distributed residuals was not met despite log X+1 transformation and removing sampling dates with zero insects present.

Because Friedman's test was performed on seasonal totals for assessing insecticide effects, the effect of sampling time and interactions between sampling time and insecticide, could not be assessed.

Supplementary Table S3. Pearson correlation between the number of egg batches and *S. litura* larvae sizes from three planting dates from 2016 trials at Bahawalpur

Larvae size	12August	21August	17 September
<1cm	0.413***	0.488***	0.147***
1-2cm	-0.64	-0.7	-0.147
>2cm	-0.143*	-0.11	-0.089

*** and * indicate significance at *P* <0.001 and *P* <0.05 respectively

Supplementary Table S4. Effect of insecticide treatments on cauliflower curd weight, marketability and revenue

Planting date	Location	Weight (g)			Marketability (%)			Revenue ($)		
		F	*df*	*P*	*F*	*df*	*P*	*F*	*df*	*P*
16th July 2015	Multan	14.50	5,12	<0.001	22.21	5,12	<0.001	20.15	5,12	<0.001
12th September 2015	Bahawalpur	2.10	5,12	0.134	14.15	5,12	<0.001	19.86	5,12	<0.001
2nd October 2015	Multan	50.16	5,12	<0.001	10.27	5,12	<0.001	6.61	5,12	0.004
11th December 2015	Bahawalpur	24.80	7,16	<0.001	48.07	7,16	<0.001	39.55	7,16	<0.001
5th January 2016	Bahawalpur	51.22	7,16	<0.001	9.59	7,16	<0.001	6.23	7,16	0.001
19th January 2016	Multan	9.62	7,16	<0.001	7.71	7,16	<0.001	5.95	7,16	0.002
12th August 2016	Bahawalpur	18.60	5,12	<0.001	33.31	5,12	<0.001	23.04	5,12	<0.001
21st August 2016	Bahawalpur	9.12	5,12	0.001	174.62	5,12	<0.001	127.01	5,12	<0.001
17th September 2016	Bahawalpur	6.77	5,12	0.003	72.52	5,12	<0.001	5.17	5,12	0.009†
2nd October 2016	Multan	45.8	5,12	<0.001	75.84	5,12	<0.001	63.12	5,12	<0.001

†Because several tests of the effects of insecticide were carried out on yield parameters we adjusted the significance criterion according to the Bonferroni procedure, dividing the significance criterion (0.05) by the number of times each parameter was evaluated: this *P*-value was no longer significant following adjustment
